# GOGrapher: A Python library for GO graph representation and analysis

**DOI:** 10.1186/1756-0500-2-122

**Published:** 2009-07-07

**Authors:** Brian Muller, Adam J Richards, Bo Jin, Xinghua Lu

**Affiliations:** 1 Department of Biostatistics, Bioinformatics and Epidemiology, Medical University of South Carolina, 135 Cannon St Suite 303 Charleston, SC 29425, USA

## Abstract

**Background:**

The Gene Ontology is the most commonly used controlled vocabulary for annotating proteins. The concepts in the ontology are organized as a directed acyclic graph, in which a node corresponds to a biological concept and a directed edge denotes the parent-child semantic relationship between a pair of terms. A large number of protein annotations further create links between proteins and their functional annotations, reflecting the contemporary knowledge about proteins and their functional relationships. This leads to a complex graph consisting of interleaved biological concepts and their associated proteins. What is needed is a simple, open source library that provides tools to not only create and view the Gene Ontology graph, but to analyze and manipulate it as well. Here we describe the development and use of GOGrapher, a Python library that can be used for the creation, analysis, manipulation, and visualization of Gene Ontology related graphs.

**Findings:**

An object-oriented approach was adopted to organize the hierarchy of the graphs types and associated classes. An Application Programming Interface is provided through which different types of graphs can be pragmatically created, manipulated, and visualized. GOGrapher has been successfully utilized in multiple research projects, e.g., a graph-based multi-label text classifier for protein annotation.

**Conclusion:**

The GOGrapher project provides a reusable programming library designed for the manipulation and analysis of Gene Ontology graphs. The library is freely available for the scientific community to use and improve.

## Introduction

Network graphs based on the Gene Ontology (GO) database are now widely used in projects that analyze biological concepts (see [1-4] and more references therein). Most published studies have utilized their own implementations of graph creation and analysis routines. The primary motivation underlying this project is to create a robust, reusable, openly distributed library for the creation, manipulation, and analysis of GO based graphs. The package can be utilized as a foundation in the future development of applications that involve the Gene Ontology using the Python computing language [5]. Python is steadily gaining in popularity within the scientific community [6-8] and we believe that this accessible programming language will continue to grow increasingly pervasive in the bioinformatics sciences [9].

### Library Description and Structure

As a reusable library, GOGrapher is a tool created primarily for the developers who write GO-related applications, so that they can reuse a broad range of common functions to save time and effort. The library contains common routines for graph operation and analysis, for instance, creation of graphs, finding shortest paths, extracting minimum spanning trees, and graph topology analysis.

**Figure 1:**
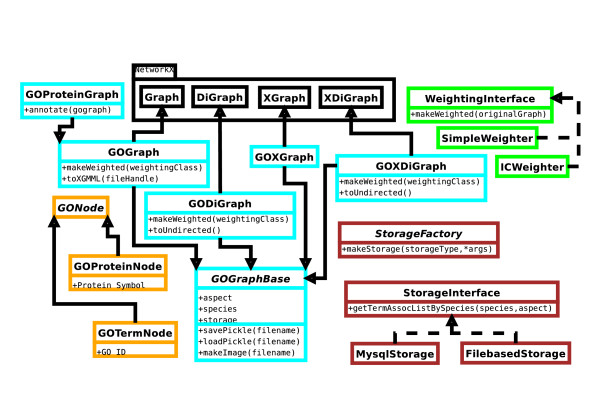
**UML Class Diagram**. This figure is a UML class diagram in which classes are grouped according to their functionalities: the *graph *classes have cyan borders; the *node *classes have orange borders; the *weighting *interfaces have green borders; and the *storage *related classes have brown borders. Dashed lines indicate that a class implements an interface (with the implementer as the source of the arrow). Solid lines indicate that a class extends the functionality of a parent class (with the extending class as the source of the arrow pointing at the parent class).

The object-oriented hierarchy of the library's classes is shown in Figure 1 as a Unified Modeling Language (UML) diagram. There are four logical groups of classes, and each is indicated by a distinct color in the figure. The first group is comprised of three classes representing the nodes (vertices) in the graphs, shown with orange borders. It includes a base *GONode *class, a term node class (*GOTermNode*), and a protein node class (*GOProteinNode*). The second group is made up of the four basic graph type classes (cyan borders) which are categorized based on weighting and directionality. The third group consists of classes that handle serialization and storage of various graph instances (shown with brown borders). Finally, a weighting interface has been created; classes that implement the interface can produce a weighted version of a graph using the weighting functions provided by users. Two example weighters are given (green borders).

#### Node Objects

Graphs are represented by collections of vertices and edges. The vertices (shown with orange borders in Fig. 1) in GOGrapher graphs represent entities such as biological concepts (GO terms) and proteins or genes.

#### Graph Objects

The GOGrapher library contains four different types of graphs (shown with cyan borders in Fig. 1) based on whether each graph is directed and whether it is weighted. A base class referred to as *GOGraphBase *is defined to represent the fundamental information about a GO ontology, e.g., which of the three aspects it represents. In addition, every graph class in this package extends a corresponding graph class from the NetworkX graphing library [8,10]. The methods and properties of the NetworkX superclasses are inherited in the GOGrapher classes. In addition, all of the graph manipulation and analysis functions [11] provided by NetworkX will work equally well for the GOGrapher graph classes. This multiple inheritance provides classes with the properties and functionalities both of a graph and of an ontology.

The definition of the GO from the Gene Ontology Consortium can be used to create an initial instance of the *GODiGraph *class (an unweighted directed graph). Other GO related graphs can be created by transforming an instance of the *GODiGraph *object into the desired type. For example, an instance of *GODiGraph *can be converted to an undirected graph, or, if given weighting information, it can be converted to a weighted graph (either directed or undirected). Since many GO terms are species specific, we allow users to specify whether or not a graph should be associated with one or more species, e.g., yeast or human, so that the terms that have never been used to annotate proteins from the species can be trimmed from the graph. The *GOProteinGraph *is a special case of an undirected, unweighted graph in which protein nodes are added to the graph with edges connecting them to their associated terms. This graph may be useful in situations in which the actual protein/term associations provide meaningful biological information.

#### Graph Creation Process

A directed unweighted graph known as a *GODiGraph *is first created according to the definition given by the Gene Ontology Consortium (see next subsection for different information sources). This graph can be further modified to produce other GO-related graphs, e.g., undirected or weighted versions, by invoking the provided method calls. Each instance of the *GODiGraph *is created with a specific aspect of the Gene Ontology, e.g., *Biological Process*. If the attribute *species *of a *GODiGraph *instance is not set, a full graph with all GO terms will be created. If a user is primarily interested in a subgraph that is relevant to a specific species, the user can associate the created GO graph with a collection of protein annotations from one or more species. Then, the leaf nodes that have not been used to annotate any proteins from the species will be trimmed. In addition, a user can create a graph in which both GO terms and proteins are first class objects, i.e., an instance of *GOProteinGraph*. In this case, a *GODiGraph *is first created according to GO definition, then the annotated proteins are added to the graph as vertices, and finally, the graph is converted into an undirected graph. This is due to the fact that there is no parent-child relationship between a GO term and a protein.

### GO Information Sources

GOGrapher provides classes (brown borders in Fig. 1) to access ontology and annotation information needed for creating GO graphs. The sources for this information are available either as a MySQL database or data files; both are available on the GO website [12].

#### Database Backend

The GOGrapher library can store all information (ontology and annotation data) needed for creating and manipulating GO-related graphs using a MySQL [13] database management system (DBMS). Current MySQL versions of the GO database are publicly provided on the Gene Ontology website [12]. To use the database as an information source, a user only needs to import the database source once, and then database access and query are performed inside GOGrapher functions and are transparent to users of library. One disadvantage with this method is the amount of time it takes to initially create graphs; interaction with a database is slower than simply reading files (the process discussed in the next section).

#### Ontology and Annotation Files

The library is also capable of using ontology definition and annotation data files from the Gene Ontology Consortium. The Web Ontology Language (OWL) representation is a suitable format for GOGrapher due to its portability and wide use. The Open Biomedical Ontology XML (OBO-XML) format is also supported by GOGrapher. The annotation files are available in a unified format. GOGrapher can instantiate a *GODiGraph *using an ontology definition file in either of the above file formats, and further association of annotated proteins to the graph can be carried out using any number of annotation files.

#### Graph Storage and Output

An instance of a GO graph class can be stored in several forms, including as an image or a serialized format. An image of a GO graph can be created and stored using the GOGrapher functions that are inherited from the PyGraphviz library [14]. All graph layouts and file formats supported by Graphviz are supported by GOGrapher. GOGrapher is also capable of exporting an extensible Graph Markup and Modeling Language (XGMML) representation [15] of GO related graphs. The format is based on the Graph Modeling Language (GML) which is widely used to describe and render graph visualization by a variety of programs, including the popular graph visualization application Cytoscape [16]. It should be noted that large graphs (for instance, a fully annotated entire GO graph) can take a long time to load in Cytoscape. Python's *cPickle *library is used for the permanent file storage and retrieval of graphs as native Python objects. It handles the serialization and unserialization of Python objects as byte streams to and from files on the user's machine.

### Graph Weighters

A weighting class that implements a set interface (green borders in Fig. 1) can be used to transform an unweighted (ether directed or undirected) graph to a corresponding weighted graph. A weighting class extends a base weighter class and must implement a single function that accepts two nodes and returns an appropriate weight according to a certain weighting scheme. It is simple for a user to implement a new weighter class that uses a different weighting process based on the weighting framework provided in GOGrapher.

### GOHeirarchicalClassifier: A Case Study

GOGrapher was also employed in a project that developed a graph-based multi-label classification system for text categorization. To train a GO graph-based classifier, an initial directed GO graph was created using GOGrapher. Using the dynamic typing capabilities of the Python language, each GO node was then associated with a classifier, e.g., a naive Bayes or a SVM classifier. The training documents were further associated with each GO term and were further propagated to their ancestor nodes, a step that utilized the topological sort functionality of NetworkX. Then the classifier associated with each node was trained using the combined documents. When given a testing document, the classifiers associated with the nodes were invoked according to the graph structure, thus the resulting multiple class labels are organized as a graph.

### Performance

GOGrapher has been tested on a number of Linux distributions (RedHat, Debian, Ubuntu) and Windows versions (Server 2003, XP). All performance tests were conducted with a graph corresponding to the biological process ontology, which contains 15,157 terms relevant to human proteins. It took under two and a half minutes to create and save a complete GO graph (including time to parse an ontology and an annotation file) on a Windows Server 2003 machine with a 2.4 GHz processor and 1 GB of RAM. A computer running Debian with a 2.4 GHz processor and 1 GB of RAM takes slightly less than two and half minutes. On both machines it takes fewer than ten seconds to simply save or load a graph.

Creating an image of a large graph can be time consuming. This expense is primarily due to the running time of the Graphviz layout algorithms. It is suggested that only images for relatively small graphs should be created. Additionally, there are currently no Windows binaries of Pygraphviz released, essentially relegating image creation to the Linux, Unix, and Mac OS X distributions. In most cases the creation of an XGMML file may be the best choice; this method is supported on all platforms. A XGMML file can be created by GOGrapher and then imported into a visualization program like the cross-platform application Cytoscape [16].

### Comparison with existing tools and future directions

To our best knowledge, GOGrapher is currently the only *programming library *available for the manipulation and analysis of the Gene Ontology graphs. Most of the other tools are distributed as independent applications, either available for download or provided as online applications, that cannot be extended to provide a framework for additional forms of analysis. GOGrapher is useful as a library upon which new tools can be built. As a library, it reduces the redundancy and complexity that come from recreating the same functionality involved in creating GO graphs, for instance, implementation of a parser for annotation files and gene ontology files. GOGrapher also provides programmers with a variety of types of network analysis functionality by the means of inheriting anther foundation library, NetworkX. As an open source library, GOGrapher library can be extended with functionalities that meet the needs of future tool developers.

## Availability and requirements

The GOGrapher library is freely available under the GNU General Public License version 3 at . The site contains a short tutorial and an online version of the API. Minimally, Python [5] and NetworkX [8,10] must be installed. For graphing functionality, Graphviz [17] and Pygraphviz [14] must also be installed. GOGrapher is cross platform software and has been tested on a variety of Linux distributions and Microsoft Windows.

## Competing interests

The authors declare that they have no competing interests.

## Authors' contributions

XL conceived the project. BM lead implementation, and all authors contributed to coding and testing of the library.
